# Green Synthesized Magnetic Nanoparticles as Effective Nanosupport for the Immobilization of Lipase: Application for the Synthesis of Lipophenols

**DOI:** 10.3390/nano11020458

**Published:** 2021-02-11

**Authors:** Renia Fotiadou, Alexandra V. Chatzikonstantinou, Mohamed Amen Hammami, Nikolaos Chalmpes, Dimitrios Moschovas, Konstantinos Spyrou, Angeliki C. Polydera, Apostolos Avgeropoulos, Dimitrios Gournis, Haralambos Stamatis

**Affiliations:** 1 Biotechnology Laboratory, Department of Biological Applications and Technologies, University of Ioannina, 45110 Ioannina, Greece; renia.fotiadou@gmail.com (R.F.); alexandra_xatzi@hotmail.com (A.V.C.); apolyder@uoi.gr (A.C.P.); 2 Department of Materials Science and Engineering, Cornell University, Ithaca, NY 14853, USA; mah424@cornell.edu; 3 Department of Materials Science and Engineering, University of Ioannina, 45110 Ioannina, Greece; chalmpesnikos@gmail.com (N.C.); dmoschov@uoi.gr (D.M.); konstantinos.spyrou1@gmail.com (K.S.); aavger@uoi.gr (A.A.); dgourni@uoi.gr (D.G.)

**Keywords:** green synthesis, *Olea europaea*, magnetic nanoparticles, lipase, immobilization, biocatalysis, bioactive lipophenols, hydroxytyrosol

## Abstract

In this work, hybrid zinc oxide–iron oxide (ZnOFe) magnetic nanoparticles were synthesized employing *Olea europaea* leaf aqueous extract as a reducing/chelating and capping medium. The resulting magnetic nanoparticles were characterized by basic spectroscopic and microscopic techniques, namely, X-ray diffraction (XRD), X-ray photoelectron spectroscopy (XPS), fourier-transform infrared (FTIR) and atomic force microscopy (AFM), exhibiting a spherical shape, average size of 15–17 nm, and a functionalized surface. Lipase from *Thermomyces lanuginosus* (TLL) was efficiently immobilized on the surface of ZnOFe nanoparticles through physical absorption. The activity of immobilized lipase was found to directly depend on the enzyme to support the mass ratio, and also demonstrated improved pH and temperature activity range compared to free lipase. Furthermore, the novel magnetic nanobiocatalyst (ZnOFe-TLL) was applied to the preparation of hydroxytyrosyl fatty acid esters, including derivatives with omega-3 fatty acids, in non-aqueous media. Conversion yields up to 90% were observed in non-polar solvents, including hydrophobic ionic liquids. Different factors affecting the biocatalyst performance were studied. ZnOFe-TLL was reutilized for eight subsequent cycles, exhibiting 90% remaining esterification activity (720 h of total operation at 50 °C). The green synthesized magnetic nanoparticles, reported here for the first time, are excellent candidates as nanosupports for the immobilization of enzymes with industrial interest, giving rise to nanobiocatalysts with elevated features.

## 1. Introduction

With the advent of nanobiotechnology, immobilization of enzymes on nanomaterials has been presented as a promising concept, giving rise to remarkable potentialities [1]. Diverse nano-scale materials (from 1 to 100 nm) have been tailored to meet the requirements as host platforms for enzymes. More specifically, nanomaterials have flourished over the past few years, ascribed to their high specific surface areas, extraordinary stability, controlled functional surfaces, intrinsic shapes and high enzyme loading [2,3,4]. As a consequence, well-designed nanobiocatalytic systems (NBSs) have been fabricated and have demonstrated essential features for scale-up exploitation; namely, elevated enzyme activity, enhanced stability over a range of parameters (temperature, pH, solvents, and storage) and remarkable reusability due to the cooperative interactions of biocatalysts with the supports. Some of them have also been efficiently applied to specific biotransformations of high added-value products. However, the production, functionalization, and purification methodologies of chemically-synthesized nanomaterials provoke major concerns regarding safety, cost-effectiveness, environmental impact and, primarily, long-term effects on human health that possibly hinder their commercialization [1,5].

Current research endeavors to introduce and exploit “green synthesis” approaches for the preparation of nanoparticles in order to tackle and compensate the inadequacies of conventional techniques such as procedure costs, insufficient yields, use of perilous reagents, generation of noxious wastes, and high energy supplies. Bio-inspired strategies have attracted great attention for the fabrication of nanomaterials, especially metal or metal oxides [5]. Natural sources such as microorganisms, plants, or even household by-products can act as a driving force for the arrangement of nano-scale materials with peculiar shapes and sizes. Biological entities are endowed with macromolecules and intriguing secondary metabolites, which can efficiently reduce metal salts to zero-valent nanoparticles (NPs) using water as the reaction medium in solely mild conditions (bottom-up approach) [5,6]. Size, shape, dispersion, yield and stability of the resulting NPs are strictly defined by physical and chemical parameters during synthesis [5,7].

Interestingly, extracts from different plant parts have proven to be more effective as reducing agents due to their potent phytochemicals, ambient and rapid protocols/synthesis, excessive production yields, and absence of cultivation or purification phases compared to microbial-derived NPs [8,9]. Plant-mediated NPs are also considered as more safe and stable formations [5,10]. Overall, phytonanotechnology can contribute to the management of bio-wastes, reinforcing sustainability and bioeconomy [9].

Furthermore, biological constituents of extracts coat the surfaces and subsequently stabilize the formulated nanomaterials, enriching them with functional groups and preventing agglomeration [8,10]. For instance, olive leaf extracts (OLEs) encompass high levels of polyphenol compounds with proven antioxidant and anti-inflammatory activities [11]. Interestingly, it has been reported that polyphenols, besides bioreduction, can chelate metal ions such as Fe^2+^, Zn^2+^, and Cu^2+^ [5,9]. Consequently, nanomaterials stemming from polyphenol-rich extracts are covered with exceptional bioactive compounds, which could enhance their potential favorable effect [5,9,12,13]. Nevertheless, the exact mechanism of bioreduction/biofrabrication has yet to be fully elucidated, but general points have been determined [5,10].

To date, certain green nanomaterials (silver, zinc oxides, copper oxides, cerium) have stood out as antimicrobial agents [10], and others (gold, ferric, platinum) have paved the way for biomedical applications [5,14,15]. On the contrary, there are scarce references of green nanomaterials used as support matrices for enzymes. Among nano-scale materials, ZnO has been recognized as safe formulations with intrinsic properties for biological applications and enzyme immobilization [16,17]. Magnetic nanoparticles (MNPs) are also nano-scale materials of utmost importance. More specifically, magnetic nanomaterials enable simple and non-laborious recovery of nanobiocatalytic systems, reinforcing recyclability [4]. Chai et al. [18] referred to the green synthesis of ZnOFe NPs as a more stable complex that also appeared to enhance antimicrobial and magnetic capacities. Due to the fact that pharmaceutical and food-related industries are now inclining to greener biosynthetic routes, plant-derived nanomaterials with elevated features could have a prominent role as immobilizing nanosupports for versatile biocatalysts of commercial interest.

Lipases (triacylglycerol acyl hydrolase, EC 3.1.1.3), which belong in the broad enzyme family of hydrolases, are significant biocatalysts, which function both in conventional and non-conventional media with high selectivity and stability, engaging in versatile applications from pharmaceuticals to biofuels. These enzymes possess a prominent role in the global market due to the ability to catalyze determinate ester synthesis in ambient conditions without unwanted by-products [19,20]. For instance, lipophenols (or phenol lipids) are biologically active compounds, consisting of a lipidic and a phenolic moiety joined with an ester bond, which have attracted marked interest among nutraceutical and pharmaceutical industries due to their beneficial cumulative or synergistic activities. Lipase-mediated production of novel lipophilic derivatives of natural phenols and polyphenols with fatty acids has received great attention over the past few years [21,22,23,24,25,26]. Hydroxytyrosol is a naturally occurring phenolic compound that derives foremost from olive tree and displays significant health benefits, especially high antioxidant activity and cardiovascular protection [11,27]. However, as a result of its high hydrophilic nature and insufficient pharmacokinetic profile, its utilization for many cosmetic to biomedical applications has been impeded [27]. The bioconjunction of hydroxytyrosol with different acyl donors, especially mono- and polyunsaturated fatty acids, increases its lipophilicity and bioavailability, whereas its antioxidant, anti-cancer, anti-inflammatory and anti-microbial activities can be also improved [23,24,26,28,29]. To date, the biosynthesis of lipophenols has been mostly attempted with commercial dispensable immobilized biocatalysts [23,28,29].

Herein, we report the green synthesis of stable magnetic ZnOFe nanoparticles from aqueous olive leaf extract, as a novel hybrid enzyme immobilizing support matrix with magnetic capabilities, a spherical shape, and functionalized surface. The newly-synthesized nanosupport was characterized by FTIR, XRD, XPS, AFM and scanning electron microscopy with energy dispersive spectroscopy (SEM/EDS) and used as an immobilizing carrier for TLL, a stereoselective lipase with numerous applications. The nanoconjugate, ZnOFe-TLL, was efficiently applied to the synthesis of hydroxytyrosyl–oleate, a biologically significant lipophenol, and other saturated or polyunsaturated fatty acid esters of hydroxytyrosol. The effect of different reaction media, the conversion rates, and the recyclability upon hydroxytyrosyl–oleate synthesis was evaluated. Moreover, the biochemical characterization of ZnOFe-TLL was conducted. To the best of our knowledge, this is the first report of an enzyme immobilized on green synthesized ZnOFe nanoparticles that effectively applied the synthesis of lipophilic derivatives of hydroxytyrosol in non-aqueous media.

## 2. Materials and Methods

### 2.1. Materials

Lipase from *Thermomyces lanuginosus* (TLL) was kindly provided from Novozymes A/S (Denmark) and was used without further purification. *p*-nitrophenol (*p*-NP), *p*-nitrophenyl butyrate (*p*-NPB), (+/−)-α-lipoic acid, myristic acid, palmitic acid, linoleic acid, methyl palmitate, methyl linoleate, dimethyl sulfoxide (DMSO) were purchased from Sigma–Aldrich (St. Louis, MO, USA); ferrous (II) sulfate heptahydrate, oleic acid, methyl oleate from Fluka, zinc acetate dihydrate from Merck (KGaA, Darmstadt, Germany); eicosapentaenoic acid (EPA) from Larodan (Solna, Sweden); and hydroxytyrosol from Carbosynth (Suzhou, China). The ionic liquids (ILs): 1-butyl-3-methylimidazolium hexafluorophsphate ([bmim][PF_6_]), 1-butyl-3-methylimidazolium tetraflfluoroborate ([bmim][BF_4_]) and methyltrioctylammonium bis(trifluoromethylsulfonyl)imide ([mtoa][NTf_2_]) were of analytical grade and purchased from Alfa-Aesar (Kandel, Germany), Fluka and Sigma–Aldrich (St. Louis, MO, USA). All organic solvents used were of analytical grade.

### 2.2. Preparation of Olive Leaf Extract

*Olea europaea* leaves, which were collected from Serres (region of Macedonia), Greece, were washed thoroughly with deionized water and shade-dried at room temperature. Then, grounded *Olea europaea* leaves in double distilled water (100 g L^−1^) were heated at 90 °C and constantly stirred for 20 min in the dark. After that period, the aqueous extract was cooled, filtrated through Whatman filter paper, and centrifuged three times at 8500 rpm for 10 min. The supernatant was stored at 4 °C under nitrogen and utilized for nanoparticles biosynthesis. Phytochemical analysis and determination of the total phenolic content of the olive leaf extract (OLE) were carried out according to standard assays [13,30,31,32].

### 2.3. Synthesis of ZnOFe Nanoparticles

*Olea europaea* leaf extract was used for the biosynthesis of ZnOFe nanoparticles as the reduction and capping medium. Biosynthesis of nanoparticles was based on previous reports with minor modifications [7,17]. An equimolar ratio of zinc acetate and ferrous sulphate (0.1 M) was dissolved in 50 mL double distilled water. Then, 1 mL of OLE was added drop wise to the mixture and constantly stirred for 1 h at 90 °C in the dark. The pH of the reaction mixture was adjusted to 12 using appropriate amounts of 2 M NaOH drop by drop. Color change of the solution from orange to dark green-brownish indicated the formation of nanoparticles. Finally, the resulting suspension was cooled and centrifuged at 8500 rpm for 20 min. The precipitate was washed twice with double distilled water and the purified green-brownish pellet was dried overnight at 37 °C. UV–Vis spectra adsorption at 350–400 nm, XRD, XPS and FTIR studies demonstrated the complete formation of ZnOFe nanoparticles. 

### 2.4. Characterization of ZnOFe Nanoparticles

UV–Vis absorption spectra of ZnOFe samples were recorded on an Agilent Cary 60 UV–Vis spectrophotometer (Agilent, Santa Clara, CA, USA) in the wavelength range 200–800 nm. Samples were diluted and dispersed in double-distilled water at room temperature.

The powder X-ray diffraction patterns were collected on a D8 Advanced Bruker diffractometer (Bruker, Billerica, MA, USA) using CuKα (40 kV, 40 mA) radiation and a secondary beam graphite monochromator. The patterns were recorded in a 2-theta (2θ) range 2 to 80°, in steps of 0.02° and counting time 2 s per step.

Atomic force microscopy (AFM) images were obtained in tapping mode with a Bruker Multimode 3D Nanoscope (Ted Pella Inc., Redding, CA, USA) using a microfabricated silicon cantilever type TAP-300G, with a tip radius of <10 nm and a force constant of approximately 20–75 N m^−1^.

X-ray photoelectron spectroscopy (XPS) measurements were performed in an ultrahigh vacuum at a base pressure of 4 × 10^−10^ mbar with a SPECS GmbH spectrometer equipped with a monochromatic MgKa source (hv = 1253.6 eV) and a Phoibos-100 hemispherical analyzer (Berlin, Germany). The spectra were collected in normal emission and energy resolution was set to 1.16 eV to minimize measuring time. Spectral analysis included a Shirley background subtraction and a peak deconvolution employing mixed Gaussian–Lorentian functions, in a least squares curve-fitting program (WinSpec) developed at the Laboratoire Interdisciplinaire de Spectroscopie Electronique, University of Namur, Belgium.

Fourier-transform infrared (FTIR) spectra was carried out in order to investigate the functionalized surface of green ZnOFe NPs and further to confirm lipase immobilization, using an FT/IR 4700 spectrometer (Jasco, Tokyo, Japan) equipped with a Peltier stabilizer DLaTGS detector. Samples were prepared in potassium bromide (KBr) pellets. All FTIR spectra were acquired in the range 400–4000 cm^−1^, at 4 cm^−1^ resolution and an average of 32 scans.

Scanning electron microscopy (SEM) images were obtained using a JEOL JSM-6510 LV SEM Microscope (Ltd., Tokyo, Japan) equipped with an X–Act EDS-detector by Oxford Instruments, Abingdon, Oxfordshire, UK (10 kV).

Transmission electron microscopy (TEM) images were obtained using an FEI Tecnai 12 BioTwin microscope. The nanoparticles were dispersed in ethanol and drop-cast on a lacy copper grid and dried for 1 h prior to the analysis.

### 2.5. Immobilization of Lipase from Thermomyces lanuginosus on ZnOFe

Immobilization of TLL was accomplished via physical adsorption onto the surface of ZnOFe nanoparticles. Briefly, 2 mg of ZnOFe NPs were dispersed in 2 mL phosphate buffer (0.025 M, pH 7.5) and sonicated for 15 min. After that, different concentrations of soluble TLL (0.5–7.5 mg mL^−1^) were added to the solution and incubated for 1 h at 30 °C, under stirring. ZnOFe-TLL nanoconjugates were recovered from the solution by applying an external magnetic field (grade N42), then washed three times with phosphate buffer (0.025 M, pH 7.5) and placed into a speed-vacuum concentrator to dry. The enzyme–NP nanocomposites were stored at 4 °C for further use. Immobilization yield was calculated according to Equation (1).

Immobilization yield = [ Ci − Cs/Ci ] × 100(1)

where Ci and Cs are enzyme concentrations in the supernatant before and after immobilization that are estimated according to Bradfords’ assay [33]. Lipase immobilization was further demonstrated by XPS, FTIR, and AFM analysis. The nanobiocatalyst is referred to as ZnOFe-TLL.

### 2.6. Enzymatic Activity of ZnOFe-TLL

The enzymatic activity of free and immobilized TLL was assayed using *p*-nitrophenyl butyrate (*p*-NPB) as the substrate. Briefly, 25 μg ZnOFe-TLL or a suitable amount of soluble TLL was added to 1 mL phosphate buffer (0.025 M, pH 7.5). The reaction was initiated with the addition of 0.5 mM *p*-NPB (diluted in DMSO). *p*-NP release was monitored at 405 nm using a UV–Vis spectrophotometer equipped with a Peltier temperature controller that maintained a constant temperature of 40 °C (Agilent, Santa Clara, CA, USA). Absorption values were taken every minute for a total time of ten minutes, and *p*-NP concentration was calculated based on a standard curve. Regarding this work, one unit of enzymatic activity (U) was defined as the amount of lipase that liberates 1 μmol of *p*-NP per minute per mL of reaction at 40 °C. Blank samples without enzymes were also prepared and no product was detected. 

### 2.7. Effect of Temperature and pH on Activity of Free and Immobilized TLL

The effect of reaction pH on the activity of free and immobilized TLL was determined over the pH range of 6 to 8.5 and at a constant temperature of 40 °C. The following buffers (25 mM) were used: potassium phosphate (pH 6.0–8.0) and Tris–HCl (pH 8.5). Enzyme activity was measured as described in Section 2.6. The effect of reaction temperature on the activity of free and immobilized TLL was carried out over the temperature range of 20 to 70 °C, with increments of 10 °C. Enzyme activity was measured based on the assay described in Section 2.6, in phosphate buffer pH 7.5, 0.025 M. The activity of free TLL at the optimal reaction pH and temperature was assigned as the 100%.

### 2.8. Enzymatic Synthesis of Hydroxytyrosyl–Fatty Acid Esters

The esterification and transesterification performance of ZnOFe-TLL nanoconjugates was evaluated on their ability to synthesize different hydroxytyrosyl–fatty acid esters. The conjunction of hydroxytyrosol with oleic acid was used as the reaction model in different anhydrous solvents (organic, ionic liquids). In a typical procedure, 0.02 mmol of hydroxytyrosol and 0.1 mmol of oleic acid were dissolved in 1 mL dehydrated solvent (n-hexane, DIPE, MTBE, acetonitrile, 2-methyl-2-butanol, t-butanol, [mtoa][NTf_2_], [bmim][PF_6_], [bmim][BF_4_]) in screw-capped flasks. After the addition of 10 mg mL^−1^ ZnOFe-TLL, the reaction mixtures were placed in an orbital shaker at 50 °C and 180 rpm. Aliquots were withdrawn at appropriate time intervals (24, 72, 120 h) to monitor the rate of each reaction. At the end of the reaction, the nanobiocatalyst was recovered using magnetic force (grade N42). 

### 2.9. High Performance Liquid Chromatography (HPLC) Analysis

All reaction aliquots were analyzed and quantified by high performance liquid chromatography (HPLC), equipped with a μBondapack C18 reversed-phase column (particle size 10 μm, length 300 mm, diameter 3.9 mm) and a diode array UV detector. The column temperature was set at 35 °C and the flow rate and injection volume were 1 mL min^−1^ and 20 μL, respectively. The mobile phase consisted of methanol (A) and water (B, with 0.1% acetic acid) with a gradient elution of 40–60% A at 0–4 min, 20–80% A at 4–8 min, 0–100% A at 8–25 min, and reset at initial conditions. Hydroxytyrosol and hydroxytyrosyl–fatty acids esters were detected at 280 nm using a photodiode array (PDA) detector. Conversion yields were determined according to Equation (2).

(2) $$Conversion\;yield\;\left(\%\right)=\frac{Total\;Area\;of\;product\;area}{Total\;Area\;of\;substrate\;area+Total\;Area\;of\;product\;peak}\;\times \;100$$

Synthesis reactions were repeated twice, while experiments in the absence of nanobiocatalyst were also conducted and hydroxytyrosyl–esters were not detected. The product of reaction model (hydroxytyrosyl–oleate) was isolated by semi-preparative HPLC, equipped with a Luna C18(2) reversed-phase column (pore size 100 Å, particle size 10 µm, length 250 mm, diameter 10 mm). The flow rate and injection volume were 5 mL min^−1^ and 2 mL, respectively. All other parameters were as described above.

### 2.10. Nuclear Magnetic Resonance (NMR) Analysis

For the characterization of the hydroxytyrosyl–oleate that was used as the reaction model, ^1^H-^13^C HSQC-HMBC NMR experiments were employed. The isolated product was dissolved in 500 μL of DMSO-d_6_, and the sample was transferred to a 5 mm NMR tube. All NMR experiments were performed on a Bruker 500 MHz AV spectrometer, equipped with a broadband inverse probe (Bruker Biospin, Rheinstetten, Germany), at 25 °C. The NMR system was controlled by the software TopSpin 3.2. The pulse sequences for ^1^H-^13^C HSQC, ^1^H-^1^3C HMBC were standard Bruker library sequences, acquired with 2 K data points over a 14 ppm spectral width.

Chemical shifts of ^1^H and ^13^C NMR (δ, ppm) of hydroxytyrosyl–oleate: ^1^H-NMR (500 MHz, DMSO-d_6_, 25 °C): δ = 0.88 (t, ^3^J_H,H_ = 7 Hz, 3H, 26c-H), 1.28 (s, 16H, 12-14c-H and 21-25c-H), 1.32 (s, 4H, 15c-H and 20c-H), 2.01 (q, ^3^J_H,H_ = 6 Hz, 4H, 16c-H and 19c-H), 2.27 (t, ^3^J_H,H_ = 8 Hz, 2H, 10c-H), 2.71 (t, ^3^J_H,H_ = 8 Hz, 2H, 7c-H), 4.13 (t, ^3^J_H,H_ = 8 Hz, 2H, 8c-H), 5.34 (t, ^3^J_H,H_ = 6 Hz, 4H, 17c-H and 18c-H), 6.47 (d, ^3^J_H,H_ = 8 Hz, 1H, 6-H), 6.63 (d, ^4^J_H,H_ = 2 Hz, 1H, 2-H), 6.66 (d, ^3^J_H,H_ = 8 Hz, 1H, 5-H), 8.65 (s,1H, 4-OH), 8.75 (s,1H, 3-OH) ppm; ^13^C-NMR (500 MHz, DMSO-d_6_, 25 °C): δ = 14.52 (C26), 25.15 (C11), 27.4 (C16,19), 29.41 (C12,13,14,21,22,23,24,25), 29.82 (C15, 20), 34.11 (C10), 34.52 (C7), 65.27 (C8), 116.11 (C5), 116,75 (C2), 119.98 (C6), 129,31 (C1), 130.31 (C17,18), 144.77 (C4), 146.05 (C3), 173.61 (C9) ppm.

### 2.11. Reusability of the Bioconjugate ZnOFe-TLL

The magnetic NBS, ZnOFe-TLL, was utilized for the constant production of hydroxytyrosyl–oleate in MTBE. Synthesis parameters are described in Section 2.8. After each cycle, the nanobiocatalytic system was reclaimed using a magnetic force (grade N42), washed with an appropriate amount of MTBE, and subsequently applied to a fresh reaction solution for ten repeated catalytic cycles (720 h of total operation). The relative activity (%) was calculated as the ratio of the remaining activity to the initial activity (first cycle).

## 3. Results and Discussion

**Scheme 1:**
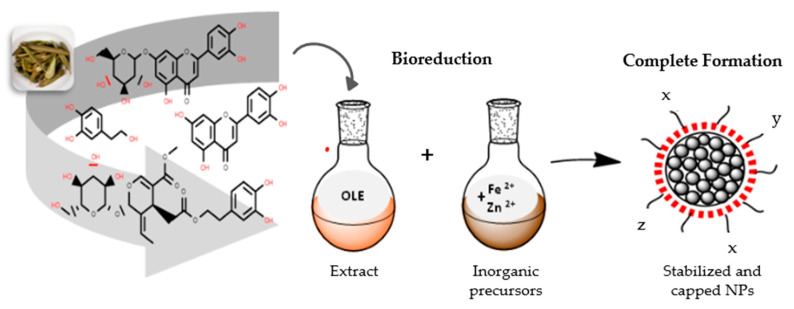
Schematic representation of ZnOFe biosynthesis from *Olea europaea* leaf extract (OLE). Phenols and flavonoids are the major constituents of aqueous OLE extract. The abundant medium served as a reducing/chelating agent for the biosynthesis of ZnOFe nanoparticles. After the bioreduction of metal ions (Zn^2+^, Fe^2+^) and growth of the resulted nanoparticles, specific compounds from the phytoconstituent-rich extract with different terminal groups (X,Y,Z: CO, COC, COOH, OH, C–C groups) act as stabilizers or capping agents, covering nanoparticles surfaces in order to prevent further agglomeration (red ring around nanoparticles).

In the present study, aqueous extract of *Olea europaea* leaves was used as the reducing and capping medium for the biofabrication of ZnOFe NPs (Scheme 1), which were further utilized as a support matrix for the immobilization of lipase from *Thermomyces lanuginosus* via physical absorption. Pristine nanoparticles and nanoparticles with the incorporation of TLL were characterized by spectroscopic and microscopic techniques. ZnOFe-TLL nanocomposites are a result of non-covalent interactions between enzyme molecules and functional groups of nanoparticles, including hydrophobic, van der Waals, electrostatic forces and hydrogen bonds [1,34]. Physical absorption is considered as a one-pot, safe, and inexpensive approach avoiding noxious linkers and preserving the enzyme microenvironment intact [1]. The novel nanobiocatalytic system was applied to synthetic reactions of compounds with biological interest in non-conventional media. 

### 3.1. Characterization of ZnOFe and ZnOFe-TLL Nanoparticles

Preliminary phytochemical screening of OLE revealed the presence of saponins, phenols (112 mg g^−1^ crude extract), flavonoids, glycosides, steroids, terpenoids, and tannins. Specific functional groups of these phytoconstituents were further confirmed by FTIR and XPS studies on the surface of ZnOFe.

ZnOFe nanoparticles were prepared and characterized by spectroscopic and microscopic techniques to investigate the morphology and the surface functionalization of the synthesized nanomaterial. UV–Vis spectra analysis revealed the characteristic absorption peak of zinc oxide at 370 nm, which is in reliance with the results in the literature and serves as an indicator for the successful formation of ZnO nanoparticles [13]. The simultaneous incorporation of ferrous sulphate to generate hybrid magnetic nanomaterials broadened the aforementioned peak. Moreover, absorption peaks at 240–250nm and 290 nm in both OLE and ZnOFe samples indicated the presence of phenolics and flavonoids (Figures S1 and S2 in the Supplementary Materials).

**Figure 1:**
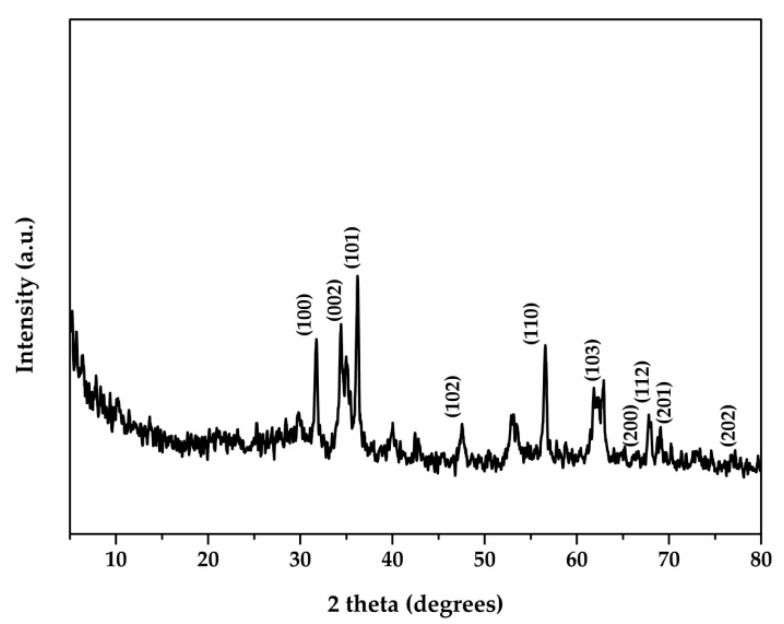
The XRD pattern of ZnOFe nanoparticles.

Powder XRD was carried out on obtained ZnOFe particles, as shown in Figure 1. The peaks observed at 2θ values of 31.7°, 34.4°, 36.2°, 47.5° 56.5°, 62.9°, 66.4°, 67.9° 69.1° and 77.1° indicated the presence of ZnO nanoparticles. These peaks can be assigned to (100), (002), (101), (102), (110), (103), (200), (112), (201) and (202) crystal planes and hexagonal crystal geometry according to ICDD card no. 00-036-1451 [35,36,37]. The peaks which appeared at 29.8°, 40°, 42.4° can be attributed to the presence of Zn(OH)_2_ (or hydroxylated ZnO NPs) phase [38,39], while the peak centered at 34.9° is assigned to the (311) Miller plane of iron oxide nanoparticles [40].

**Figure 2:**
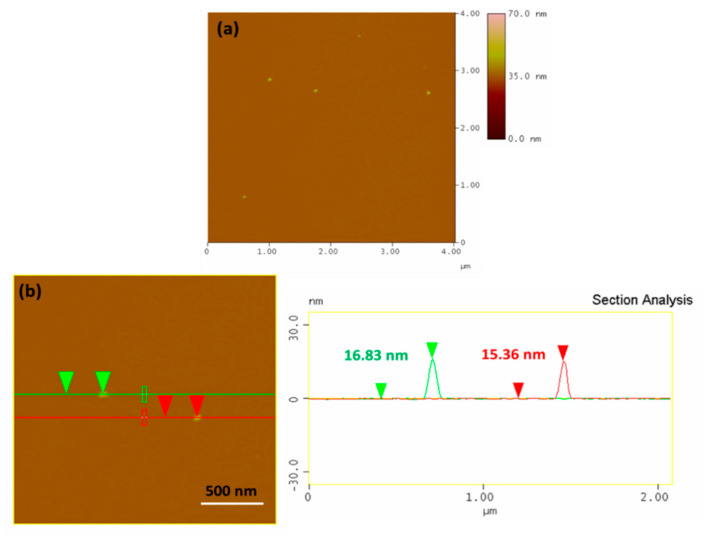
Representative atomic force microscopy (AFM) height images (**a**) and cross section analysis profile (**b**) of ZnOFe nanoparticles deposited on Si-wafers by drop-casting from aqueous dispersions.

**Figure 3:**
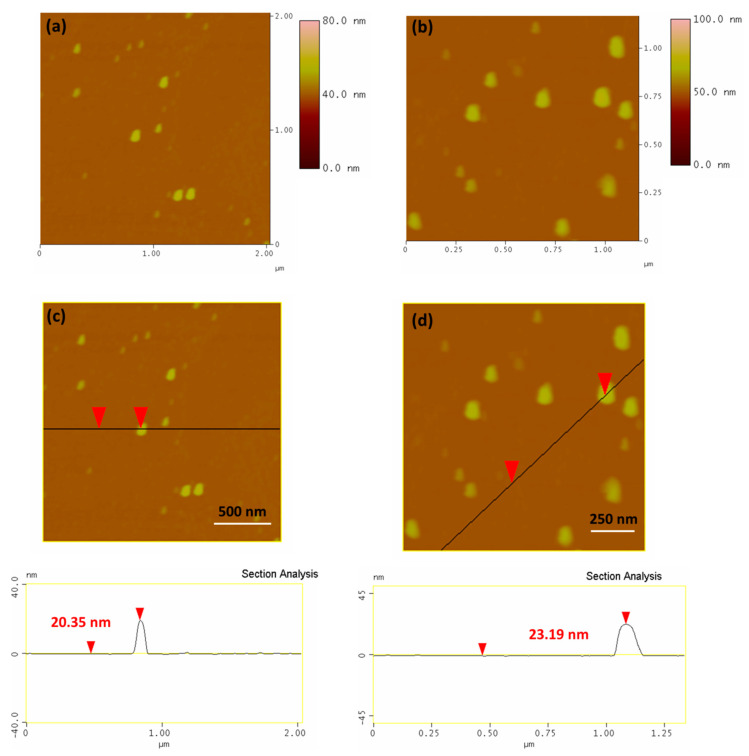
AFM height (**a**,**b**) and cross sectional analysis (**c**,**d**) images of ZnOFe-enzyme nanoparticles deposited on Si-wafers by drop-casting from aqueous dispersions.

The structure and the morphology of the ZnOFe nanoparticles as well as the ZnOFe-TLL nanoparticles were revealed by atomic force microscopy (AFM). Representative AFM images of both systems deposited on Si-wafers by drop-casting from aqueous dispersions are presented in Figure 2 and Figure 3, respectively, confirming the presence of nanoparticles. In addition, the depiction of cross section analysis image of the ZnOFe nanoparticles is shown in Figure 2b and revealed nanoparticles with an average size of 15–17 nm. On the other hand, AFM height images of ZnOFe-TLL nanoparticles are presented in Figure 3a,b. From these images, it is clear that after the incorporation of the enzyme, the spherical shape is slightly disrupted. Moreover, from the cross-sectional analysis AFM images (Figure 3c,d) the average size of the nanoparticles has been increased due to the binding of the enzyme with a value in the range of 20–24 nm. These findings are in totally accordance with the TEM study. TEM portraits also revealed a slightly disrupted circular nanostructure due to the immobilization of the enzyme (Figure S3).

**Figure 4:**
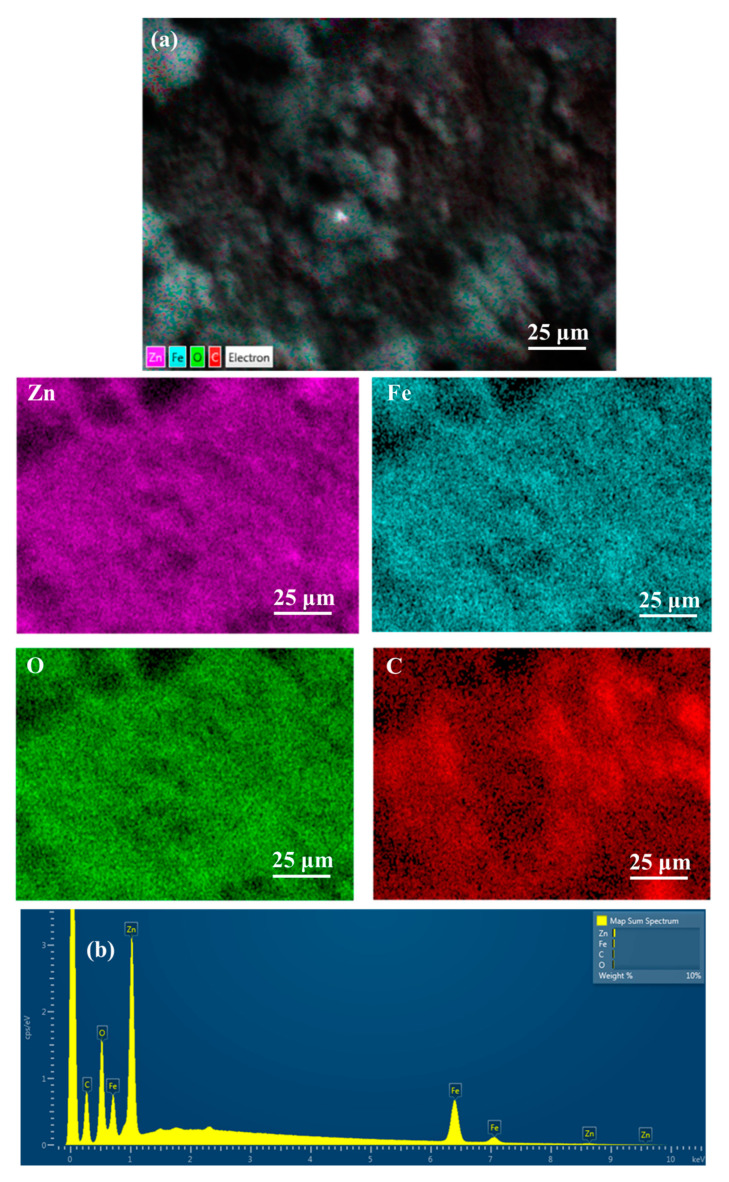
Representative elemental mapping of ZnOFe nanoparticles (Zn, Fe, O, C), (**a**) scanning electron microscopy micrograph of sample in the same region, (**b**) EDS analysis.

Scanning electron microscopy and energy-dispersive X-ray spectroscopy were employed to confirm the composition of ZnOFe nanoparticles and the effective metal loadings in the hybrid materials. Figure 4 shows representative SEM/EDS mapping images and the elemental analysis of ZnOFe nanoparticles. Images depict the EDS elemental analysis with colors, including zinc, iron, oxygen, and carbon elements, which was also ascertained by the XPS survey (Figure S4). The composition of the hybrid magnetic nanoparticles was estimated to 40.3 wt.% Zn, 25.8 wt.% Fe, 16.2 wt.% O, and 17.7 wt.% C.

**Figure 5:**
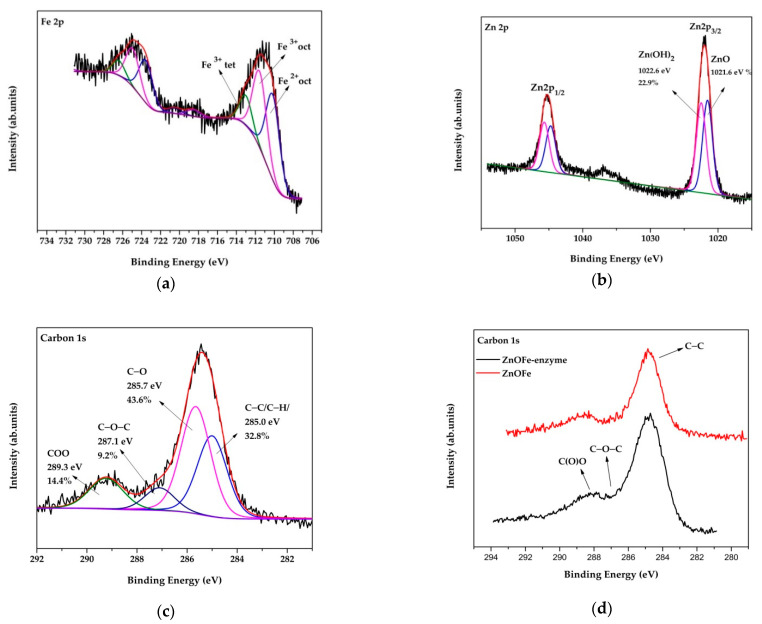
XPS spectrum of (**a**): Fe2p, (**b**): Zn2p, (**c**): C1s Zn–iron oxide nanoparticles. (**d**): Comparative C1s spectrum of Zn–iron oxide nanoparticles after the enzyme immobilization.

XPS of ZnOFe revealed the different oxygen states of Fe and Zn. More specifically, after fitting in the Fe2p3/2 high resolution photoelectron spectrum presented in Figure 5a, Fe^2+^ states were observed in lower binding energy due to octahedral formations and Fe^3+^ states in higher binding energies owing to octahedral and tetrahedral formation. Furthermore, zinc exists in two oxidation states (Figure 5b). The high energy peak at 1022.6 eV is attributed to Zn^2+^ in the form of Zn(OH)_2_ [41,42], as revealed in the Zn2p3/2 photoelectron spectrum, while the fitted peak at lower energy (1021.6 eV) is ascribed to the ZnO chemical state [43]. Accordingly, in order to attain additional information for the functional groups of the resulting nanoparticles, the C1s photoelectron peak was evaluated. The peak is deconvoluted into four fitted peaks (Figure 5c) [44], creating in such way a highly functional material. In detail at 285.0 eV, the presence of C–C and C–H bonds represents 32.8% of the whole carbon amount, while a highly intense peak at 285.7 eV is due to C–O functionalities. Two additional peaks at 287.1 and 289.3 eV arise due to C–O–C and C(O)O functionalities, representing 9.2% and 14.4% of the whole carbon amount, respectively.

Based on the XRD pattern, there is no sign of the presence of Fe-doped ZnO nanoparticles due to the coexistence of the diffraction peaks of both phases and without any shift of the reflections of the ZnO phase (that could be an indication of a doped material). Hence, this is a hybrid material where zinc and iron coexist as oxide nanoparticles. Moreover, the magnetic properties of these materials were proved by simply applying a commercial magnet (Figure S5).

From the comparative C1s photoelectron spectrum of ZnOFe nanoparticles before and after the enzyme incorporation, the existence of C–O–C (approximately 286.9 eV) as well as amide bonds (approximately 288.0 eV, Figure 5d) reveals the successful immobilization of lipase on the nanoparticles surface.

**Figure 6:**
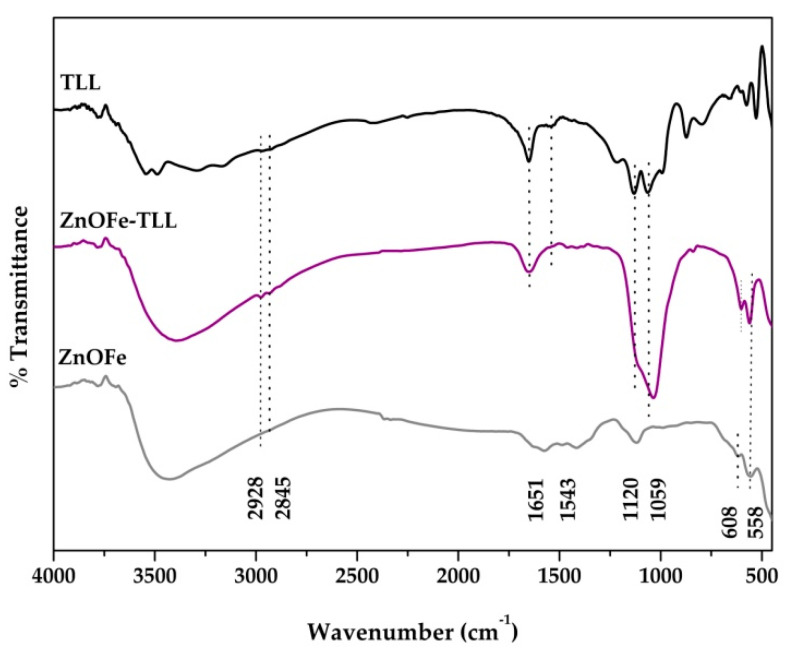
FTIR spectra of ZnOFe-TLL compared to: pristine ZnOFe NPs and soluble TLL.

To further ascertain the immobilization of TLL, FTIR spectra of ZnOFe-TLL were investigated in the range of 400–4000 cm^−1^ and compared with those of soluble TLL and pristine nanoparticles from OLE (Figure 6). The FTIR spectrum of ZnOFe showed several peaks, implying the presence of functional groups from OLE, at 2928, 2845, 1632, 1571, 1478, 1119, 608, 558 and 460 cm^−1^. The weak bands located at 2928 and 2845 could be assigned to asymmetric and symmetric stretching vibrations of alkyl groups [45,46]. The peak observed at 1632 cm^−1^ corresponds to stretching vibrations of C=O groups, and the peaks located at 1571, 1487 and 1413 cm^−1^ could be assigned to stretching vibrations of C=C and C–C aromatic groups [18,46]. The peak obtained at 1120 cm^−1^ indicates the presence of C–O–C epoxy groups and those at 608, 558, and 460 cm^−1^ represent typical absorption peaks of metal oxides owing to Zn–O and Fe–O bonds [18,47,48]. The incorporation of lipase generated characteristic peaks which confirm the immobilization of TLL on the surface of ZnOFe NPs. Specifically, the peak at 1651 cm^−1^ clearly indicates stretching vibrations of C=O group of the peptide bond, and the weak peak at 1543 cm^−1^ refers to bending vibrations of N–H groups. The broad peak in the range 950–1250 cm^−1^ corresponds to stretching vibrations of C–N, C–O (primary and secondary alcohols), and strong bending vibrations of C-H groups [18,46,48]. The detection of the aforementioned peaks in the ZnOFe-TLL spectrum correlates with those of TLL and pristine ZnOFe nanoparticles and demonstrates that OLE acted as a chelating and capping medium, and TLL was successfully anchored on the surface of ZnOFe. Certain typical peaks of phytochemicals were not detected or shifted on the ZnOFe-TLL spectrum due to the non-covalent interactions developed between the functional groups of the nanocarrier and the biocatalyst.

### 3.2. Immobilization Yield and Biocatalytic Characteristics of Immobilized TLL

**Figure 7:**
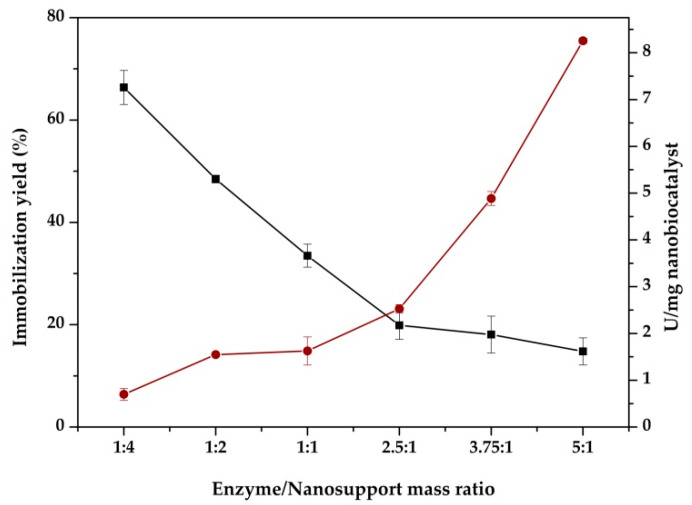
Effect of enzyme to nanosupport mass ratio on immobilized TLL activity (●) and immobilization yield (▪). One unit of enzymatic activity (U) was defined as the amount of lipase that liberates 1 μmol of *p*-NP per minute per mL of reaction at 40 °C.

The biochemical characteristics of immobilized lipase were evaluated based on the hydrolytic activity of the enzyme. Figure 7 illustrates the immobilization yield and the hydrolytic activity of immobilized TLL in correlation with the enzyme to support the (E/S) mass ratio. The amount of ZnOFe NPs (2 mg) used for immobilization was constant in the overall process. It was observed that the immobilization yield was decreased almost proportionally with the increase in the E/S mass ratio. Sarno et al. [49] also exhibited that the amount of TLL during immobilization on Fe_3_O_4_ NPs had a profound effect on the immobilization efficiency, presenting a similar pattern with the present work. Dantas et al. [50], who reported the immobilization of TLL on nonporous polystyrene particles through physical absorption, achieved immobilization yields that did not exceed 62%. The immobilization yield of TLL on ZnOFe surfaces reached 69%, when the enzyme to support mass ratio was 1:4. The aforementioned results confirm a dependency between E/S mass ratio and immobilization yield, which is commonly observed [51]. When lower E/S proportions were used, higher immobilization yields were accomplished, and vice versa. 

Increase in the initial protein content (or E/S ratio) led to increased lipase adsorption (loading enzyme) on the surface of ZnOFe nanoparticles (Table S1). Lage et al. also pointed out that initial TLL content affected several features, including immobilization yield, total loaded protein, and hydrolytic activity, in a similar manner with this study [52]. Regarding the activity of immobilized TLL, a positive linear relationship with E/S mass ratio increase was observed. The hydrolytic activity of immobilized TLL was proportionally enhanced due to higher amounts of bound enzyme on the surface of ZnOFe NPs, which is in accordance to that observed for the immobilization of other enzymes onto various nanomaterials [45,51,53].

### 3.3. Effect of Reaction Temperature and pH on the Activity of Free and Immobilized TLL

**Figure 8:**
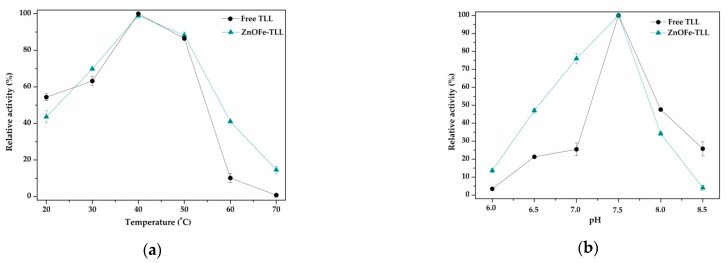
(**a**) Effect of temperature on the hydrolytic activity of free (●) and immobilized TLL (▲) at pH 7.5; (**b**) Effect of pH on the hydrolytic activity of free (●) and immobilized TLL (▲) at 40 °C. The activity of the free enzyme on the optimal temperature and the optimal pH was taken as 100%.

The hydrolytic activity of free and immobilized TLL was determined at various temperatures, ranging from 20 to 70 °C in phosphate buffer 25 mM, pH 7.5. Figure 8a shows that both free and immobilized biocatalyst exhibited a similar temperature pattern, while the temperature optimum (40 °C) of TLL was not shifted due to enzyme immobilization. At low incubation temperature (20 °C), the relative hydrolytic activity of the free TLL was slightly higher than the activity of the immobilized enzyme. Above 50 °C, the relative activity of free enzyme dramatically declined, whereas the relative activity of ZnOFe-TLL was three-fold higher at 60 °C and 18-fold at 70 °C compared to the free enzyme, indicating that TLL immobilization on ZnOFe nanoparticles stabilized the enzyme. Improved activity of immobilized lipase at elevated temperatures is considered as a major advantage for large scale applications, indicating better temperature adaptability. Studies have reported a similar pattern regarding the immobilization of TLL on different nanomaterials, such as magnetic [54] and ZnO-coated nanoparticles [55].

The impact of pH on the hydrolytic activity of free and immobilized TLL was also examined. Figure 8b shows that the optimum operating pH of lipase was 7.5 in both cases. Furthermore, immobilized TLL exhibited significantly higher relative activity in acidic and neutral pH, whereas free TLL showed better performance in alkaline solutions. The decrease in the relative activity of ZnOFe-TLL in alkaline solution could be attributed to the specific microenvironment (charge effects) that the functionalized nanocarrier forms around enzyme’s active center [56,57]. However, it is obvious that soluble TLL is more susceptible to pH fluctuations displaying a narrow activity range, whereas the immobilization of lipase substantially improved its performance.

Hence, it is obvious that physical absorption of TLL on the ZnOFe surface enhances the operational activity of lipase, expanding its temperature and pH action amplitude which can be attributed to the stabilization of enzyme conformation on the surface of magnetic nanoparticles in a similar manner to that reported by Sarno et al. [49] for the immobilization of TLL on magnetic nanoparticles. Moreover, the stabilization of TLL observed after its immobilization on green synthesized ZnOFe is in accordance with the increased stabilization reported for other enzymes immobilized on nanoparticles [45,58,59]. 

### 3.4. Αcylation of Hydroxytyrosol with Fatty Acids Catalyzed by ZnOFe-TLL

ΤLL is considered as a useful biocatalyst of industrial interest because it has been utilized for the preparation of various esters for pharmaceutical and nutraceutical applications [49,50,52]. In this work, immobilized TLL on ZnOFe NPs was applied to the synthesis of lipophilic derivatives of hydroxytyrosol through its esterification or transesterification reaction with various fatty acids or fatty acid methyl esters (FAMEs), respectively. Table 1 summarizes the conversion yields of the enzymatic modification of hydroxytyrosol catalyzed by ZnOFe-TLL in MTBE. Generally, the acyl chain length of fatty acids has been reported to affect the conversion yield of the esterification reaction. Moreover, saturated and unsaturated fatty acids differ in their spatial configuration, affecting their bio-modification [28]. In the present work, it was observed that as the acyl chain length of fatty acids was increased, the conversion yield slightly decreased, which is in accordance with previous work [25,60]. A similar pattern was also noticed in the case of fatty acids with increased number of double bonds. Moreover, when FAMEs were introduced as acyl donors, conversion yields were slightly lower compared to free forms of fatty acids that were used as acyl donors. Methanol, generated as a by-product of the transesterification reaction, could act as inhibitor, slightly impairing enzyme performance or competing with the substrates for the active center of lipase [61,62,63].

**Figure 9:**
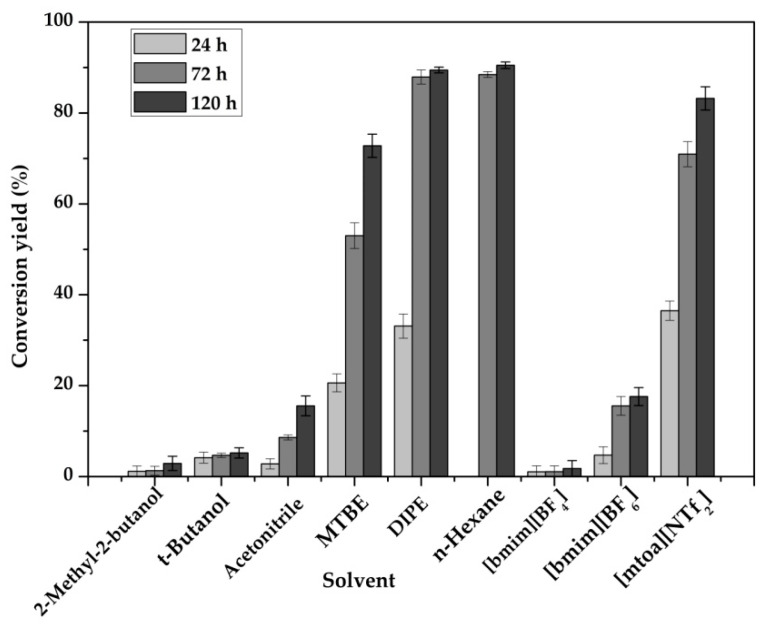
Effect of solvent and reaction time on the bioconversion of hydroxytyrosol with oleic acid catalyzed by ZnOFe-TLL at 50 °C and 180 rpm.

In order to investigate the ability of ZnOFe-TLL to catalyze lipophilization reactions in various non aqueous media, the conjunction of hydroxytyrosol with oleic acid was used as the reaction model. Synthesis of hydroxytyrosyl–oleate (Scheme S1) was confirmed by 2D ^1^H–^13^C HSQC and ^1^H–^13^C HMBC NMR analyses. The ^1^H and ^13^C chemical shift assignments of the product hydroxytyrosyl–oleate are presented in Section 2.10. of the Materials and Methods. Various hydrophilic or hydrophobic organic solvents and ionic liquids were tested as reaction media. Furthermore, the production rate of hydroxytyrosyl–oleate was monitored on each solvent and the results are presented in Table 2. All experiments were conducted at 50 °C with vigorous stirring which led to reduced mass transfer limitations. As shown in Figure 9, the conversion yields of hydroxytyrosol, catalyzed by ZnOFe-TLL, was up to 90% in the case of non-polar solvent mediated reactions, namely in n-hexane, DIPE and MTBE. Specifically, the conversion yield achieved in n-hexane was 89% in 72 h. Among ethers, it was observed that the rate and yield of esterification reaction was higher in DIPE. In contrast, immobilized TLL-mediated synthesis in polar solvents led to low yields of esterification. Among them, the conversion yield in acetonitrile reached a maximum of 20%. Reaction rates were also significantly higher in non-polar media (Table 2). More specifically, the rate of reaction in n-hexane was 34-fold higher compared to the more polar acetonitrile. Generally, non-polar/hydrophobic solvents tend to shift lipase conformation from closed to open, which has a major impact on their activity [64]. Furthermore, essential water molecules covering enzyme surface are not disrupted in non-polar media, whereas in polar solvents, this water layer is interrupted, destabilizing lipases conformation [64,65,66]. More specifically, it has been reported that polar solvents displace water molecules at the more polar regions of enzymes [65]. As a consequence, the structural characteristics and dynamic properties of biocatalysts are significantly affected. Moreover, Kumar et al. has reported that thermostable lipases, such as TLL, are more susceptible to be inactivated in water miscible organic solvents [64]. It is also interesting to note that the higher conversion yields observed in hydrophobic solvents (n-Hexane, DIPE or [mtoa][NTf_2_],) could be explained by solvent effects on solvation of hydrophilic hydroxytyrosol in a similar manner as described for other enzymatic transformations [67,68].

The ability of ZnOFe-TLL to catalyze the lipophilization of hydroxytyrosol was also investigated in ionic liquids that are frequently used as “green” solvents [66]. The highest conversion yield and reaction rate was observed in [mtoa][NTf_2_] (Figure 9, Table 2), a water immiscible IL, in which hydroxytyrosol solubility was the lowest among tested ILs [69]. Interestingly, [mtoa][NTf_2_] was proved considerably effective as a reaction medium for the lipophilization of hydroxytyrosol, despite the fact that it is a more viscous IL (574cP) than [bmim][PF_6_] (460cP) and [bmim][BF_4_] (219cP). Generally, high viscosity leads to mass transfer limitations; however, it has been reported that proteins could be stabilized in such media [66]. It was also proposed that low solubility of a substrate in ILs is related to weak interactions between substrate and solvent that could increase the availability of the substrate to the enzyme microenvironment, and, thus, increase the effectiveness of the biocatalytic process [70]. The successful employment of ZnOFe-TLL in [mtoa][NTf_2_] confirms that this nanobiocatalyst could successfully carry out synthetic reactions not only in organic but also in ionic solvents.

Furthermore, the ability of ZnOFe-TLL to catalyze the synthesis of high value hybrid bioactive compounds, such as esters of hydroxytyrosol with omega-3 fatty acids, was also investigated. For that reason, esterification of eicosapentaenoic acid (EPA) with hydroxytysosol was tested in organic solvents such as MTBE, DIPE and n-hexane. As shown in Table 3, the highest conversion yield was observed for the conjunction of EPA with hydroxytyrosol (80% after 72 h) in the less polar solvent (n-hexane), which is in accordance with that observed for the conjugation of hydroxytyrosol with oleic acid, as has been described before. However, conversion yields with the polyunsaturated fatty acid as acyl donor were slightly lower. As has been reported, unsaturated FAs tend to bend (Table S2), hampering the advance of reaction, due to steric hindrance effects [25,60]. The number of double bonds has a direct correlation with the molecular structure of the fatty acid which, as a consequence, affects the advance of acylation reaction.

### 3.5. Reusability of the Nanobiocatalytic System ZnOFe-TLL

**Figure 10:**
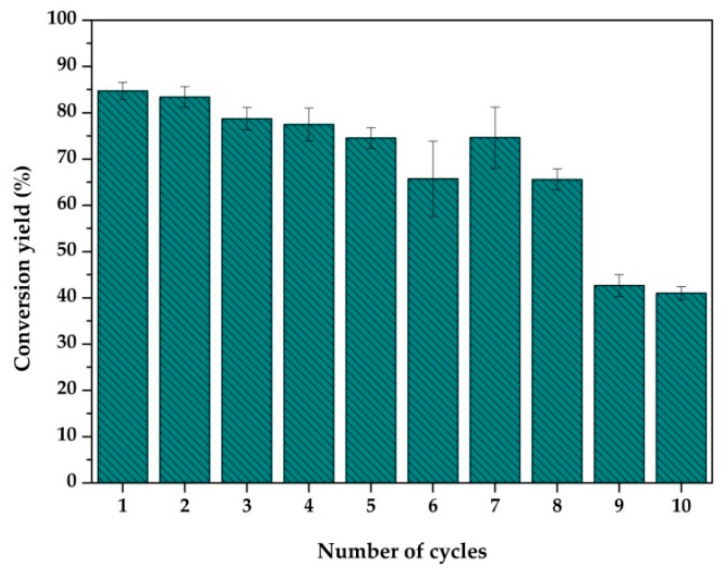
Operational stability of ZnOFe-TLL towards the consistent synthesis of hydroxytyrosyl-oleate in MTBE (15 mg mL^−1^, 72 h per cycle). The nanobiocatalytic system was retrieved after its cycle by applying a commercial magnet.

The cost of soluble lipases and, more importantly, the inability of their recovery, has impeded their deployment for many scale-up applications. These bottlenecks have been overcome by the emergence of immobilization. More specifically, enzymes immobilized on nanoparticles with magnetic capabilities can easily be recovered by magnetization, avoiding laborious isolation. Furthermore, the lipase-mediated synthesis of attractive compounds requires not only the reutilization of the nanobiocatalytic system, but also sufficient catalytic performance for a number of cycles. Hence, ZnOFe-TLL recovery and reusability upon hydroxytyrosyl–oleate synthesis in MTBE was investigated. After its cycle, the nanobiocatalyst was retrieved by magnetization (grade N42, Figure S5), washed with a proper amount of solvent, and applied to a new reaction mixture, avoiding laborious isolation by magnetization. As shown in Figure 10, immobilized lipase retained almost 90% of its initial activity after eight subsequent cycles, whereas it operated for 720 h at 50 °C displaying significantly high remaining activity. Bonazza et al. also reutilized TLL immobilized on modified chitosan support (50 mg mL^−1^) for five successful reaction cycles with a minimal loss of activity for the production of ethyl oleate [71]. The high operational stability of ZnOFe-TLL in anhydrous media, observed here, indicates that this novel nanobiocatalyst could be successfully used in various continuous biocatalytic processes of industrial interest.

## 4. Conclusions

Green synthesized hybrid magnetic ZnOFe nanoparticles were proven excellent candidates as support matrices for the immobilization of a lipase with numerous industrial applications. An eco-friendly and inexpensive methodology was employed both for the benign fabrication of hybrid magnetic nanoparticles and their conjugation with TLL. ZnOFe exhibited sufficient magnetic capabilities with simple recovery, a functionalized surface, and an average size of 15–17 nm. The exploitation of the nanobiocatalytic system, ZnOFe-TLL, for the synthesis of bioactive hydroxytyrosyl fatty acid esters, such as hydroxytyrosyl–oleate or eicosapentaenoate, led to high production yields in different non-conventional media, including ionic liquids. Furthermore, the operational activity and stability of lipase was substantially enhanced upon immobilization. These results indicate that ZnOFe-TLL is a robust candidate for many biocatalytic processes in aqueous and non-aqueous media. To the best of our knowledge, this is the first time that green ZnOFe NPs have been synthesized from olive leaf extract and used as an enzyme immobilization carrier. This captivating concept of bio-synthesized nanomaterials, along with biocatalysis, is a key solution for industrial operations, fulfilling the fundamental requisites of more environmentally friendly and safer processes. The development of green nanosupport matrices for enzyme immobilization purposes is a nascent and economically viable approach, conforming to the new era of green biotechnology.

## Figures and Tables

**Table 1:** Effect of different acyl donors on the conversion yield of hydroxytyrosol acylation catalyzed by ZnOFe-TLL after 72 h of incubation in MTBE.

Acyl Donor	Conversion Yield (%)
Lipoic acid (C8:0)	63.7 ± 2.3
Myristic acid (C14:0)	64.3 ± 1.4
Palmitic acid (C16:0)	57.1 ± 3.1
Oleic acid (C18:1)	55.0 ± 1.9
Linoleic acid (C18:2)	50.4 ± 1.1
Methyl palmitate	52.2 ± 0.8
Methyl oleate	43.1 ± 4.5
Methyl linoleate	48.3 ± 0.4
Eicosapentaenoic acid (C20:5)	12.1 ± 2.6

**Table 2:** Reaction rates (mM h^−1^ g^−1^ nanobiocatalyst) of hydroxytyrosol acylation with oleic acid catalyzed by ZnOFe-TLL in various non-aqueous media.

Solvents	Rate (mM h^−1^ g^−1^ Nanobiocatalyst)
2-Methyl-2-butanol	0.75
Acetonitrile	3.44
n-Hexane	25.31
MTBE	15.45
DIPE	24.33
t-Butanol	1.54
[bmim][PF_6_]	3.33
[bmim][BF_4_]	1.25
[mtoa][NTf_2_]	23.27

**Table 3:** Effect of reaction medium on the esterification of eicosapentaenoic acid (EPA) with hydroxytyrosol in 50 °C and 72 h of incubation.

Solvent	Acyl Donor	Conversion Yield (%)
MTBE	EPA	12.1 ± 2.6
DIPE	EPA	44.5 ± 1.6
n-Hexane	EPA	80 ± 0.9

## Supplementary Materials

The following are available online at https://www.mdpi.com/2079-4991/11/2/458/s1. Figure S1. UV–Vis absorption spectrum of aqueous OLE, ZnOFe nanoparticles and metal ions solution without extract (RT, 15 μg mL^−1^); Figure S2. UV–Vis absorption spectrum of aqueous ZnOFe nanoparticles and ZnOFe-TLL (RT, 15 μg mL^−1^); Figure S3. Representative TEM images of ZnOFe nanoparticles with the incorporation of the enzyme, TLL; Figure S4. XPS survey of ZnOFe hybrid nanoparticles; Figure S5. Magnetization of the ZnOFe NPs in (**a**) aqueous solution and (**b**) powder phase by a N42 magnet; Table S1. Enzyme loading per mg support and enzyme activity of the nanobiocatalytic system at 40 °C and pH 7.5, using *p*-NPB as the substrate; Scheme S1. Enzymatic synthesis of hydroxytyrosyl–oleate catalyzed by ZnOFe-TLL in MTBE, 50 °C and 72 h of incubation. The product was characterized by ^1^H-^13^C HSQC and ^1^H-^1^3C HMBC NMR; Table S2. Structures of saturated and unsaturated fatty acids utilized as acyl donors for hydroxytyrosol bioconversion by ZnOFe-TLL. The number of double bonds and the effect on the molecular structure are depicted in the table.
