# Supplementary material for: Green Synthesized Magnetic Nanoparticles as Effective Nanosupport for the Immobilization of Lipase: Application for the Synthesis of Lipophenols
Source: Nanomaterials (Basel). 2021 Feb 11;11(2):458. doi: 10.3390/nano11020458 (PMC7916844; doi:10.3390/nano11020458)
Supplement: Supplementary file 1 [file nanomaterials-11-00458-s001.pdf]

nanomaterials

Supplementary Material

Green synthesized magnetic nanoparticles as effective nanosupport for the immobilization of lipase: Application for the synthesis of lipophenols

Renia Fotiadou [1], Alexandra V. Chatzikonstantinou [1], Mohamed Amen Hammami [3], Nikolaos Chalmpes [2], Dimitrios Moschovas [2], Konstantinos Spyrou [2], Angeliki C. Polydera [1], Apostolos Avgeropoulos [2], Dimitrios Gournis [2] and Haralambos Stamatis [1,*]

[1] Biotechnology Laboratory, Department of Biological Applications and Technologies, University of Ioannina, 45110 Ioannina, Greece; renia.fotiadou@gmail.com (R.F.); alexandra_xatzi@hotmail.com (A.V.C.), apolyder@uoi.gr (A.C.P.)
[2] Department of Materials Science and Engineering, University of Ioannina, 45110 Ioannina, Greece; chalmpesnikos@gmail.com (N.C.); dmoschov@uoi.gr (D.M.); konstantinos.spyrou1@gmail.com (K.S.); aavger@uoi.gr (A.A.); dgourni@uoi.gr (D.G.)
[3] Department of Materials Science and Engineering, Cornell University, Ithaca, NY 14853, USA; mah424@cornell.edu (MAH)
* Correspondence: hstamati@uoi.gr ; Tel.: +30-265-100-7116

Received: date; Accepted: date; Published: date

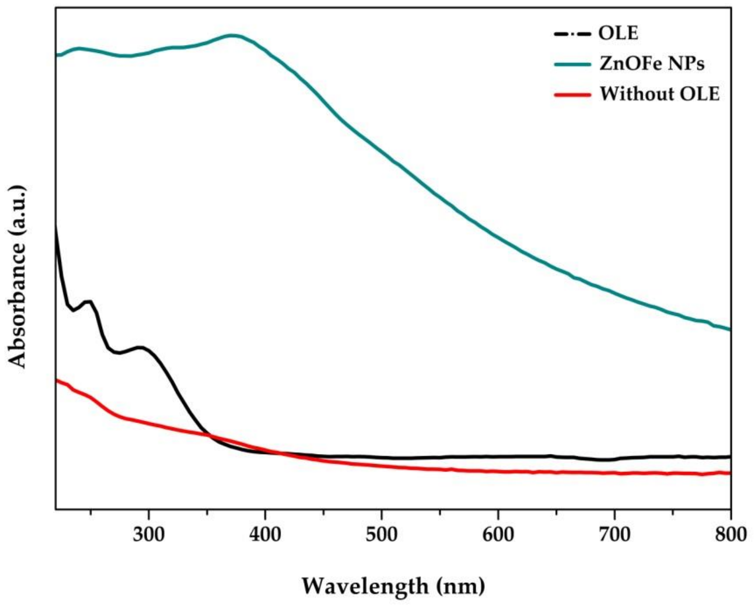

Figure S1. UV-Vis absorption spectrum of aqueous OLE, ZnOFe nanoparticles and metal ions solution without extract (RT, 15μg mL^{-1})

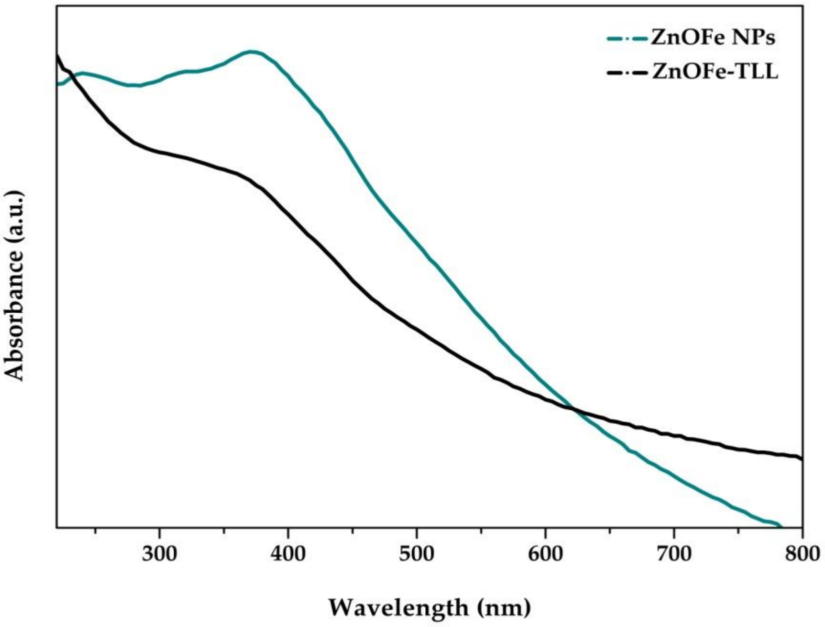

Figure S2. UV-Vis absorption spectrum of aqueous ZnOFe nanoparticles and ZnOFe-TLL (RT, 15μg mL^{-1})

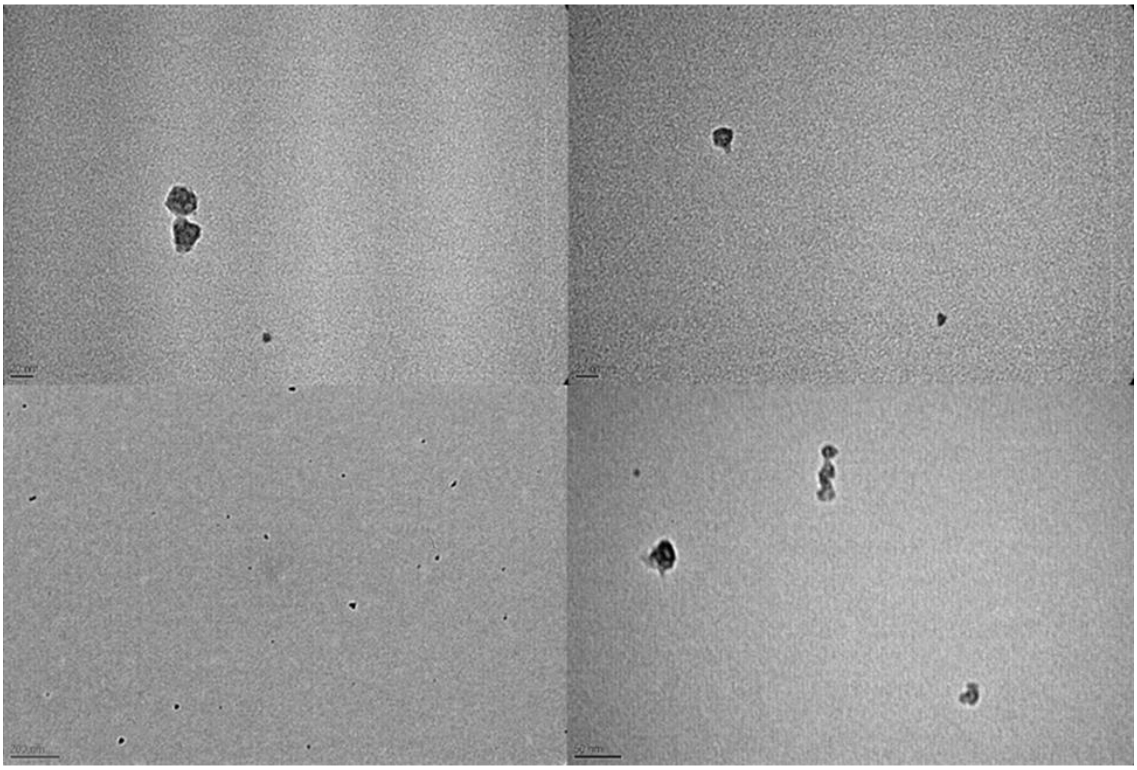

Figure S3. Representative TEM images of ZnOFe nanoparticles with the incorporation of the enzyme, TLL

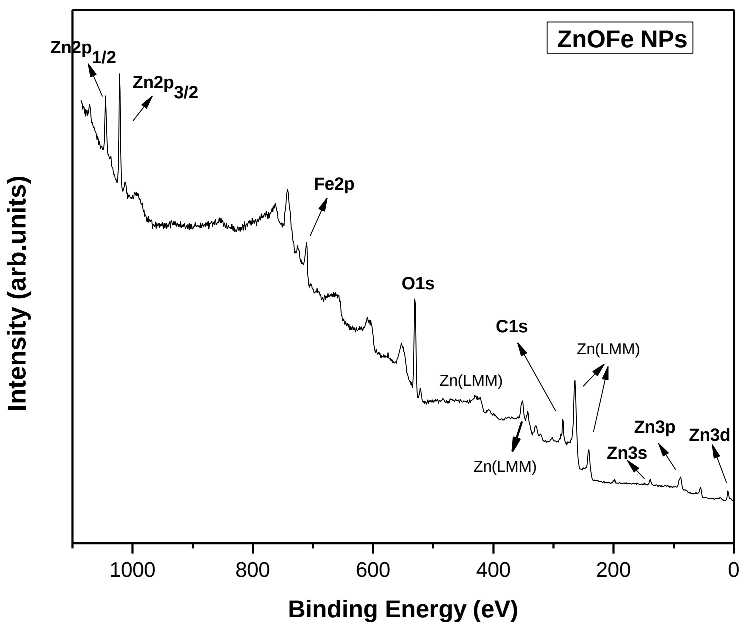

Figure S4. XPS survey of ZnOFe hybrid nanoparticles

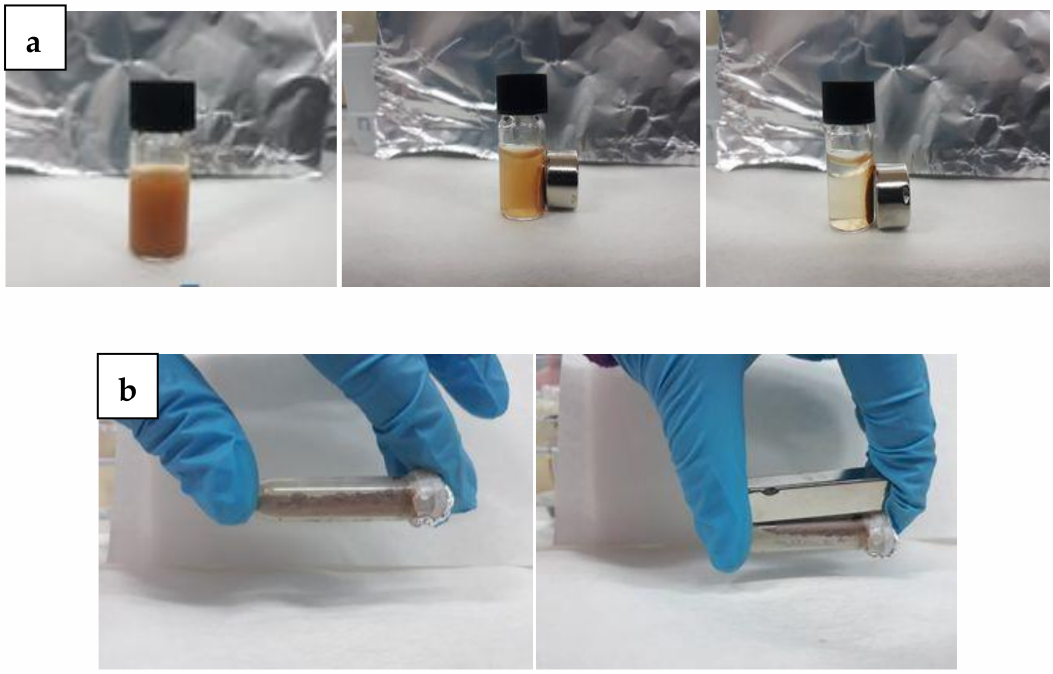

Figure S5. Magnetization of the ZnOFe NPs in a) aqueous solution and b) powder phase by a N42 magnet

Table S1. Enzyme loading per mg support and enzyme activity of the nanobiocatalytic system at 40ºC and pH 7.5, using *p*-NPB as the substrate.

Initial enzyme concentration (mg)	U mg^{-1} nanobiocatalyst	Enzyme loading mg^{-1} nanosupport
0.5	0,699	0,170
1	1,546	0,240
2,5	1,829	0,308
3,75	2,150	0,403
5	2,528	0,450
6,25	3,650	0,578
7,5	4,885	0,600
10	8,221	1,01

One unit of enzymatic activity (U) was defined as the amount of lipase that liberates 1 μmol of *p*-NP per minute per mL of reaction at 40ºC.

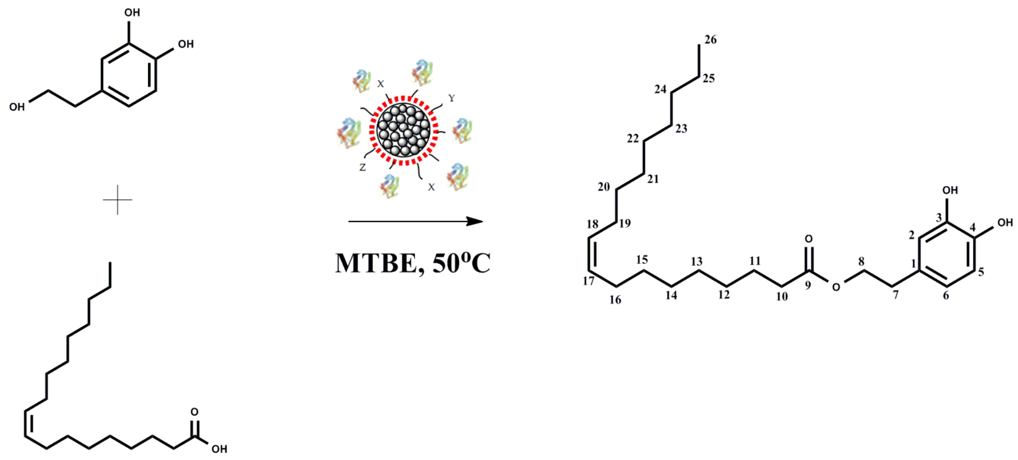

Scheme S1. Enzymatic synthesis of Hydroxytyrosyl Oleate catalyzed by ZnOFe-TLL in MTBE, 50ºC and 72h of incubation. The product was characterized by ^{1}H-^{13}C HSQC and ^{1}H-^{13}C HMBC NMR.

Table S2. Structures of saturated and unsaturated fatty acids utilized as acyl donors for hydroxytyrosol bioconversion by ZnOFe-TLL. The number of double bonds and the effect on the molecular structure are depicted in the Table.

Fatty acid	Acyl chain	Structure
Lipoic acid	C8:0	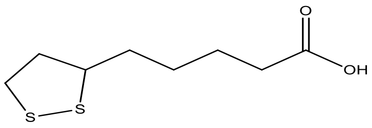
Myristic acid	C14:0	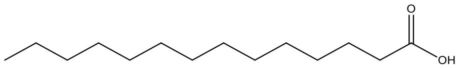
Palmitic acid	C16:0	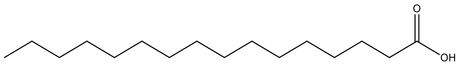
Oleic acid	C18:1	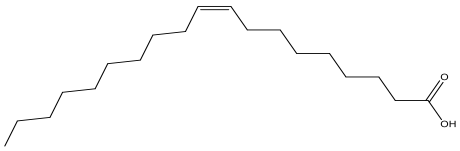
Linoleic acid	C18:2	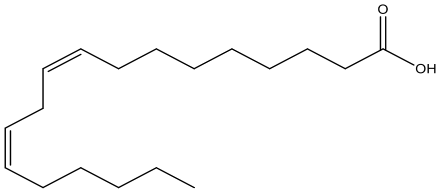
Eicosapentaenoic acid	C20:5	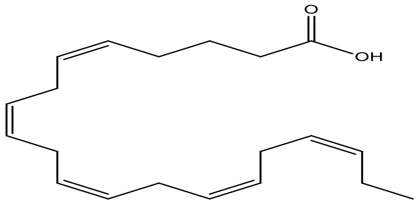

© 2020 by the authors. Submitted for possible open access publication under the terms and conditions of the Creative Commons Attribution (CC BY) license (http://creativecommons.org/licenses/by/4.0/).
